# Isolation and Molecular Characteristics of a Novel Recombinant Avian Orthoreovirus From Chickens in China

**DOI:** 10.3389/fvets.2021.771755

**Published:** 2021-12-07

**Authors:** Jun Zhang, Tuofan Li, Weikang Wang, Quan Xie, Zhimin Wan, Aijian Qin, Jianqiang Ye, Hongxia Shao

**Affiliations:** ^1^Ministry of Education Key Laboratory for Avian Preventive Medicine, Key Laboratory of Jiangsu Preventive Veterinary Medicine, Yangzhou University, Yangzhou, China; ^2^Jiangsu Co-innovation Center for Prevention and Control of Important Animal Infectious Diseases and Zoonoses, Yangzhou, China; ^3^Institutes of Agricultural Science and Technology Development, Yangzhou University, Yangzhou, China; ^4^Joint International Research Laboratory of Agriculture and Agri-Product Safety, The Ministry of Education of China, Yangzhou University, Yangzhou, China

**Keywords:** avian orthoreovirus, isolation, genomic characteristics, phylogenetic analysis, recombination, σC protein

## Abstract

In recent years, the emergence of avian orthoreovirus (ARV) has caused significant losses to the poultry industry worldwide. In this study, a novel ARV isolate, designated as AHZJ19, was isolated and identified from domestic chicken with viral arthritis syndrome in China. AHZJ19 can cause typical syncytial cytopathic effect in the chicken hepatocellular carcinoma cell line, LMH. High-throughput sequencing using Illumina technology revealed that the genome size of AHZJ19 is about 23,230 bp, which codes 12 major proteins. Phylogenetic tree analysis found that AHZJ19 was possibly originated from a recombination among Hungarian strains, North American strains, and Chinese strains based on the sequences of the 12 proteins. Notably, the σC protein of AHZJ19 shared only about 50% homology with that of the vaccine strains S1133 and 1733, which also significantly differed from other reported Chinese ARV strains. The isolation and molecular characteristics of AHZJ19 provided novel insights into the molecular epidemiology of ARV and laid the foundation for developing efficient strategies for control of ARV in China.

## Introduction

Avian orthoreovirus (ARV) belongs to the *Orthoreovirus* genus (*Reoviridae* family) with a double-stranded and segmented RNA genome (1, 2). According to electrophoretic mobility, ARV has 10 genomic segments including the large genomic segments (L1 to L3), the medium genomic segments (M1 to M3), and the small genomic segments (S1 to S4), which encode 12 major proteins (1–4). Among them, σC protein, a component of the outer capsid of the virion, encoded by S1 segment, is the main immunogenic protein and the viral cell attachment protein that induces specific neutralizing antibodies (3, 5, 6). Based on the genetic characteristics of σC protein, ARV isolates are divided into four to six clusters (7–11). ARV was initially discovered as the causative agents of tenosynovitis in chicks (12), and subsequently spread globally to multiple hosts including turkeys, pheasants, Muscovy ducks, geese, and other wild birds (13–15). ARV infection mainly causes arthritis syndrome and runting–stunting syndrome, and is also related to hepatitis, myocarditis, osteoporosis, respiratory diseases, and even central nervous system (16–18). However, only the direct link of ARV infection with viral arthritis syndrome has been conclusively confirmed (19). Notably, ARV infection results in immunosuppression, poor production performance, poor feed utilization, and conversion rate. ARV infection also increases the susceptibility to other pathogens (20–22).

Although vaccines have been used to prevent and control the diseases caused by ARV, the frequent emergence of novel ARV strains with variant antigenicity is challenging the current vaccine strategy (7, 8, 23–25). Thus, it is critical to clarify the prevalence and variant characteristics of ARV strains. In this study, a novel ARV strain, designated as AHZJ19, was isolated from a chicken flock with viral arthritis syndrome using LMH cells, and its complete genome sequence was determined. Sequence analysis revealed that AHZJ19 was significantly different from the ARVs previously isolated in China, and is a novel recombinant ARV originated from Hungarian strains, North American strains, and Chinese strains.

## Materials and Methods

### Samples

In 2019, two liver tissue samples were collected from chickens on a farm in Anhui, China. The native chickens were all 90-day-old and showed lameness, viral arthritis, and poor production performance. The liver tissue samples were homogenized with phosphate-buffered saline (PBS) and supernatants were collected by centrifugation at 12,000 rpm for 30 min at 4°C. Then, the collected supernatants were filtered through 0.22-μm filters and used for virus isolation.

### Reverse Transcription Polymerase Chain Reaction

The total RNA was extracted from the liver tissue sample suspension using the AxyPrep Multisource RNA Miniprep Kit (Axygen, USA) and cDNA was synthesized using the HiScript II 1st Strand cDNA Synthesis Kit (Vazyme, China). For detection of ARV, the PCR was performed with specific primers of S2 gene listed in Table 1 (26). The PCR reaction volume was 25 μl containing 12.5 μl of 2× Taq PCR Master Mix (Vazyme, China), 1 μl of each primer, 9.5 μl of double-distilled water (ddH_2_O), and 1 μl of the cDNA template. The PCR cycling conditions for the S2 gene amplifications were as follows: 1 cycle of 94°C for 5 min, 35 cycles of 94°C for 1 min, 55°C for 1 min and 72°C for 1 min, followed by a final extension step of 72°C for 10 min. In addition, PCR detection for chicken infectious anemia virus (CIAV), reticuloendotheliosis virus (REV), A subgroup of avian leukosis virus (ALV-A), B subgroup of avian leukosis virus (ALV-B), J subgroup of avian leukosis virus (ALV-J), and K subgroup of avian leukosis virus (ALV-K) was performed. The primers used are listed in Table 2.

**Table 1:** Primers for RT-PCR detection of S2 gene of ARV.

**Name**	**Primer sequence (5^′^-3^′^)**	**Length (bp)**
ARV-S2-PF	CCCATGGCAACGATTTC	399
ARV-S2-PR	TTCGGCCACGTCTCAAC	

**Table 2:** Primers for PCR detection of CIAV, REV, ALV-A, ALV-B, ALV-J, and ALV-K.

**Name**	**Sequence (5^′^-3^′^)**	**Length (bp)**	**Reference**
CIAV-PF	ATGAACGCTCTCCAAGAAGATAC	366	(27)
CIAV-PR	TTACAGTCTTATACGCCTTTTTGCG		
REV-PF	TGAGGGAAAATGTCATGCAACATCC	204	(28)
REV-PR	ATCCCTACCCCACCCAGTAG		
ALV-A-PF	ACCCGGAGAAGACACCCTT	563	(29)
ALV-A-PR	AGGGGTGTCTAAGGAGAAACCG		
ALV-B-PF	ACCCGGAGAAGACACCCTT	563	
ALV-B-PR	CTGGGTCGGTCAGAAGGATGT		
ALV-J-PF	ACCCGGAGAAGACACCCTT	563	
ALV-J-PR	CATAGGGCCTTATAAGAAGGTCAT		
ALV-K-PF	ACCCGGAGAAGACACCCTT	559	
ALV-K-PR	TATAGCGGAGGAGGAGCCACCTCGT		

### Virus Isolation and Identification

The supernatants of the RT-PCR-positive liver tissue samples were inoculated into LMH cells for virus isolation and the cells were incubated at 37°C with 5% CO_2_. The supernatants were collected at 36–48 h post infection (hpi) by centrifugation at 12,000 rpm for 15 min at 4°C and passaged every 2 days. The supernatants of LMH cells after seven consecutive blind passages were all tested by RT-PCR.

### Sequence and Phylogenetic Analysis

The total RNA of the isolate was sequenced using the high-throughput sequencing (Illumina sequencing technology: Illumina Novaseq 6000, ABclonal Whole RNA-seq Lib Prep kit, NovaSeq S4 flowcell) by Shanghai Tanpu Biological Technology Co. (Shanghai, China), and the sequences were assembled with *de novo* SPAdes assembly software, LASTZ, and SAMtools commands (10, 30, 31). The GenBank accession numbers for the obtained sequences of the isolate are listed in Table 3. The sequences of 12 major proteins of the isolate were aligned with the reference strains deposited in GenBank using the ClustalW methods in Megalign program by the Lasergene 7.0 software. The phylogenetic tree was constructed by using the neighbor-joining method in MEGA6.1 software with 1,000 bootstrap replicates and the intra-segmental recombination detection was performed by Bootscan analysis within the Simplot program version 3.5.1, using the neighbor-joining method, with a Kimura 2-parameter applied and 100 replicates.

**Table 3:** The GenBank accession numbers for the obtained sequences of AHZJ19.

**AHZJ19 genes**	**GenBank accession numbers**
Segment L1	OK077993
Segment L2	OK077994
Segment L3	OK077995
Segment M1	OK077996
Segment M2	OK077997
Segment M3	OK077998
Segment S1	OK077999
Segment S2	OK077802
Segment S3	OK077803
Segment S4	OK077804

### Serum Preparation and Indirect Immunofluorescence Assay

Six 3-day-old specific-pathogen-free (SPF) chicks were inoculated with 200 μl of the isolate each through unilateral food pad. Sera from the infected chickens were collected at 3 weeks post inoculation (wpi), and the antibodies against the isolate were detected by indirect immunofluorescence assay (IFA). Briefly, the infected LMH cells were fixed with pre-chilled acetone-ethanol (3:2) for 5 min. After washing once with PBS, the cells were incubated with chicken sera against the isolate (1:200) for 45 min at 37°C. Then, the cells were washed with PBS three times followed by incubation with FITC-labeled rabbit anti-chicken IgG (1:150) for 45 min at 37°C. After three washes with PBS, the cells were observed under a fluorescence microscope and the viral titer of the isolate was calculated by the Reed-Muench method (32).

## Results and Discussion

The disease of ARV has become endemic in China since its first report in 1985 (33). Although the mortality caused by ARV is not high, the infection of ARV generally results in immunosuppression, affects the normal growth and production performance of broilers, and increases the susceptibility to other pathogens. Notably, the continuous emergence of ARV mutants in recent years have caused huge economic losses to the poultry industry (8, 24, 25).

**Figure 1:**
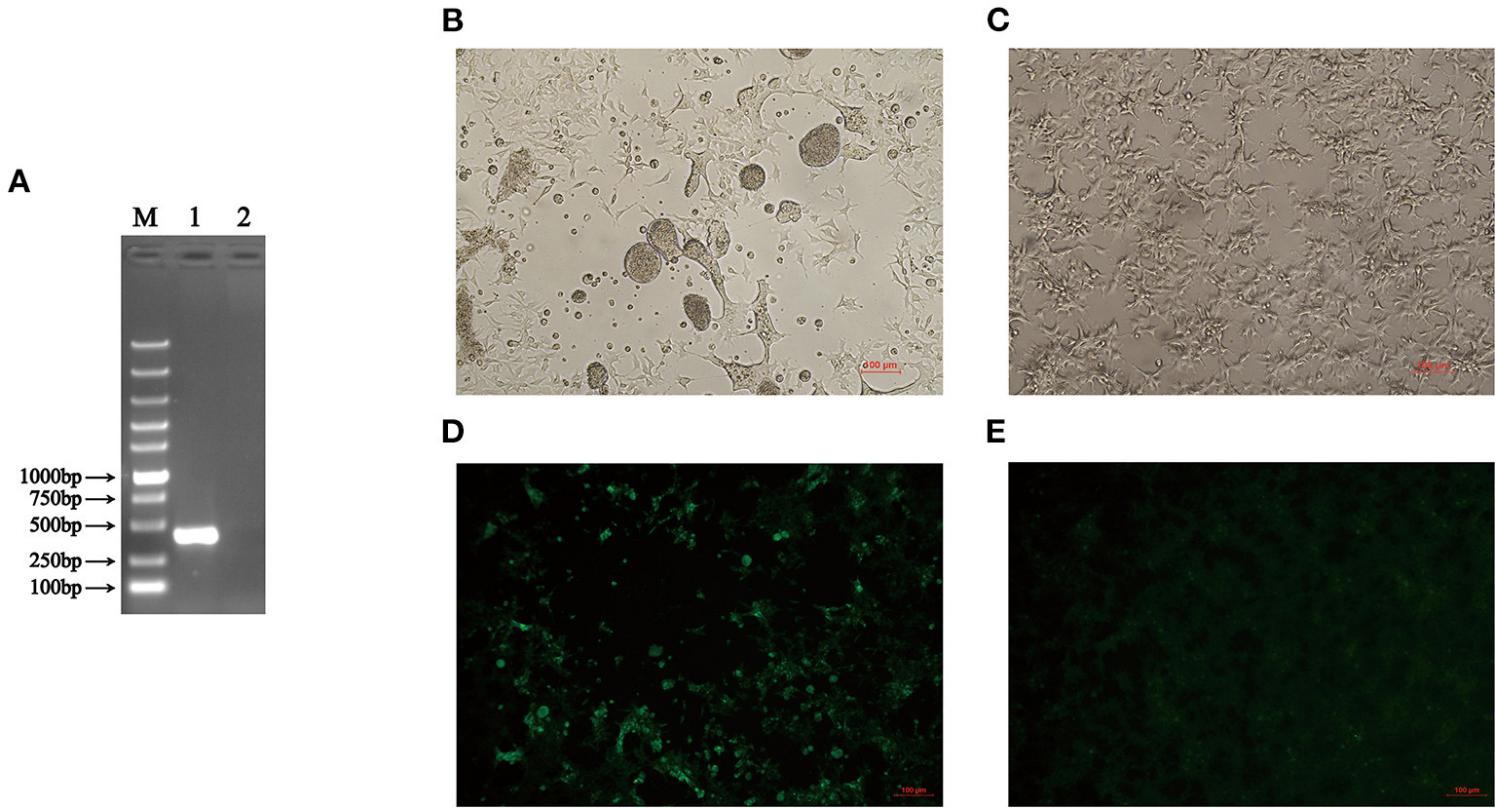
Isolation and identification of AHZJ19. **(A)** RT-PCR detection of ARV. The total RNA extracted from the liver tissue samples was tested by RT-PCR using primers specific to ARV. Lane M was Super DNA Marker; Lane 1 was the liver tissue of chickens from that farm; Lane 2 was negative control. **(B,C)** The CPE in LMH cells at 24 hpi after inoculation with supernatants of the RT-PCR-positive liver tissue sample. **(B)** The supernatants of the RT-PCR-positive liver tissue sample were inoculated into LMH cells and the cells showed the typical characteristic syncytial lesions at 24 hpi. **(C)** The negative LMH cells were considered as control. **(D,E)** IFA for detecting antibodies against ARV in chicken serum from chicks inoculated with AHZJ19. The chicken serum collected and prepared at 3 wpi was used for detected the LMH cells by IFA. **(D)** LMH cells infected with AHZJ19 at 24 hpi. **(E)** Negative LMH cells was considered as control.

In 2019, an indigenous chicken flock in Anhui in China showed clinical symptoms of viral arthritis syndrome, hepatitis, and stunting/malabsorption syndrome. To detect whether the chickens in that farm were infected with ARV, the liver tissue samples were collected from clinically ill chickens. Total RNA was extracted from the supernatant obtained from homogenized liver tissue samples. The ARV detection was performed by RT-PCR using specific primers listed in Table 1. The liver tissue was positive in the RT-PCR assay for ARV (Figure 1A). In addition, the sample was free of CIAV, REV, ALV-A, ALV-B, ALV-J, and ALV-K infection by PCR using specific primers listed in Table 2. The results of the RT-PCR detection and the sequence of the PCR product (data not shown) confirmed that the chickens were infected with ARV. To further isolate the ARV, the supernatants of the RT-PCR-positive liver tissue samples were inoculated into LMH cells and the supernatants were blindly passaged every 2 days. The infected cells showed typical characteristic of syncytial cytopathic effect at 24 hpi (Figures 1B,C) and the RT-PCR detection of the supernatant of LMH cells after 7 passages was also ARV positive. All these demonstrated that a chicken origin ARV is efficiently isolated and designated as AHZJ19. Moreover, the chicken sera specific to AHZJ19 was generated by the inoculation of AHZJ19 in 3-day-old SPF chicks through unilateral foot pad. As shown in Figure 1D, the prepared chicken sera against AHZJ19 efficiently reacted with the LMH cells infected with AHZJ19, but not with the negative control cells (Figure 1E). In addition, we found that the isolate AHZJ19 could replicate efficiently in LMH cells and the virus titer of AHZJ19 could reach 8.7 × 10^8^ TCID_50_/ml.

**Figure 2:**
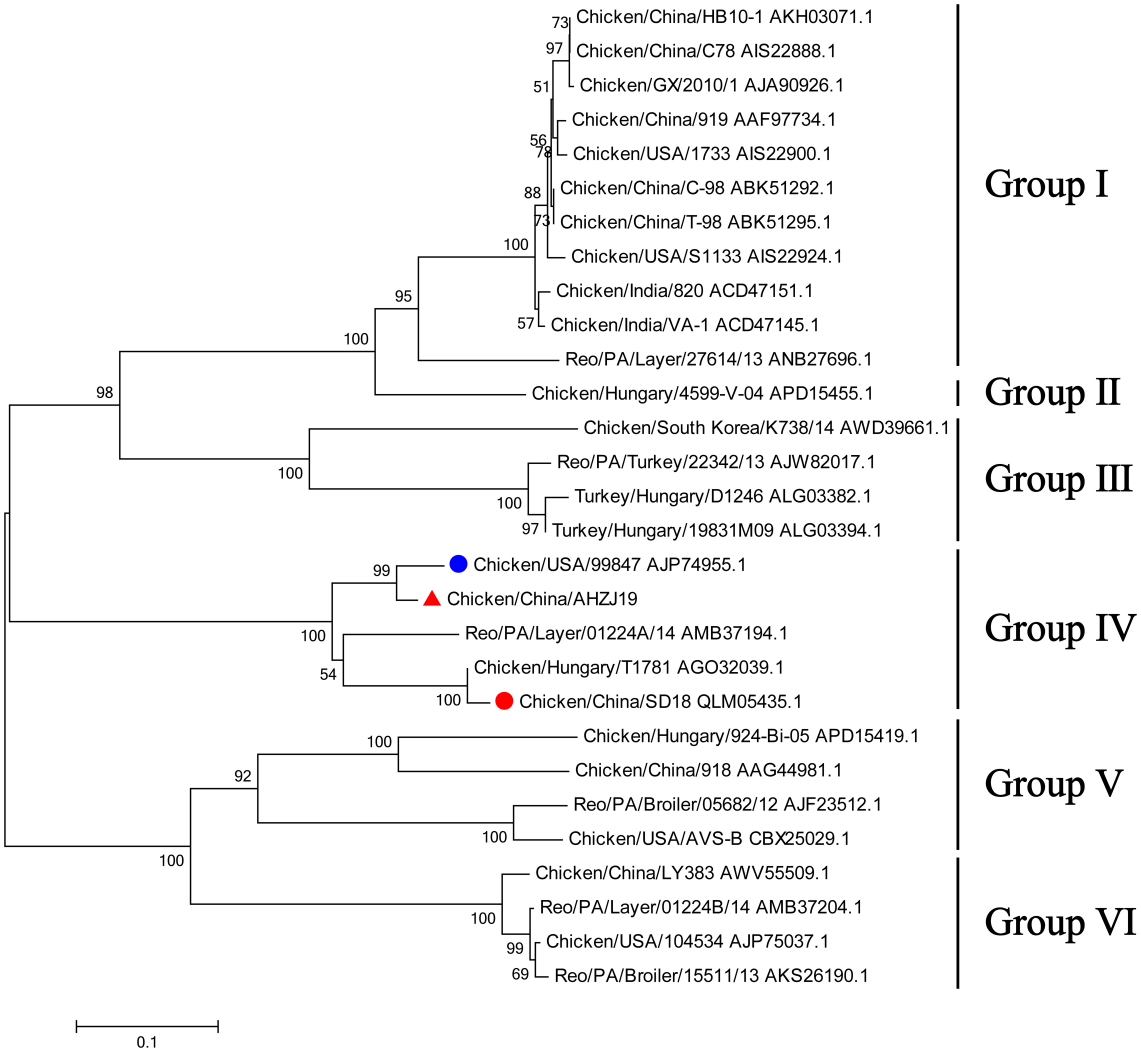
Phylogenetic tree of AHZJ19 and reference strains based on aa sequences of σC protein. The phylogenetic tree was constructed using the neighbor-joining method (1,000 bootstraps) with MEGA6. The AHZJ19 strain isolated in Anhui in 2019 is indicated by the red triangle. The strain sharing the highest homology with AHZJ19 in the world and in China is indicated by the blue and red circle, respectively.

**Figure 3:**
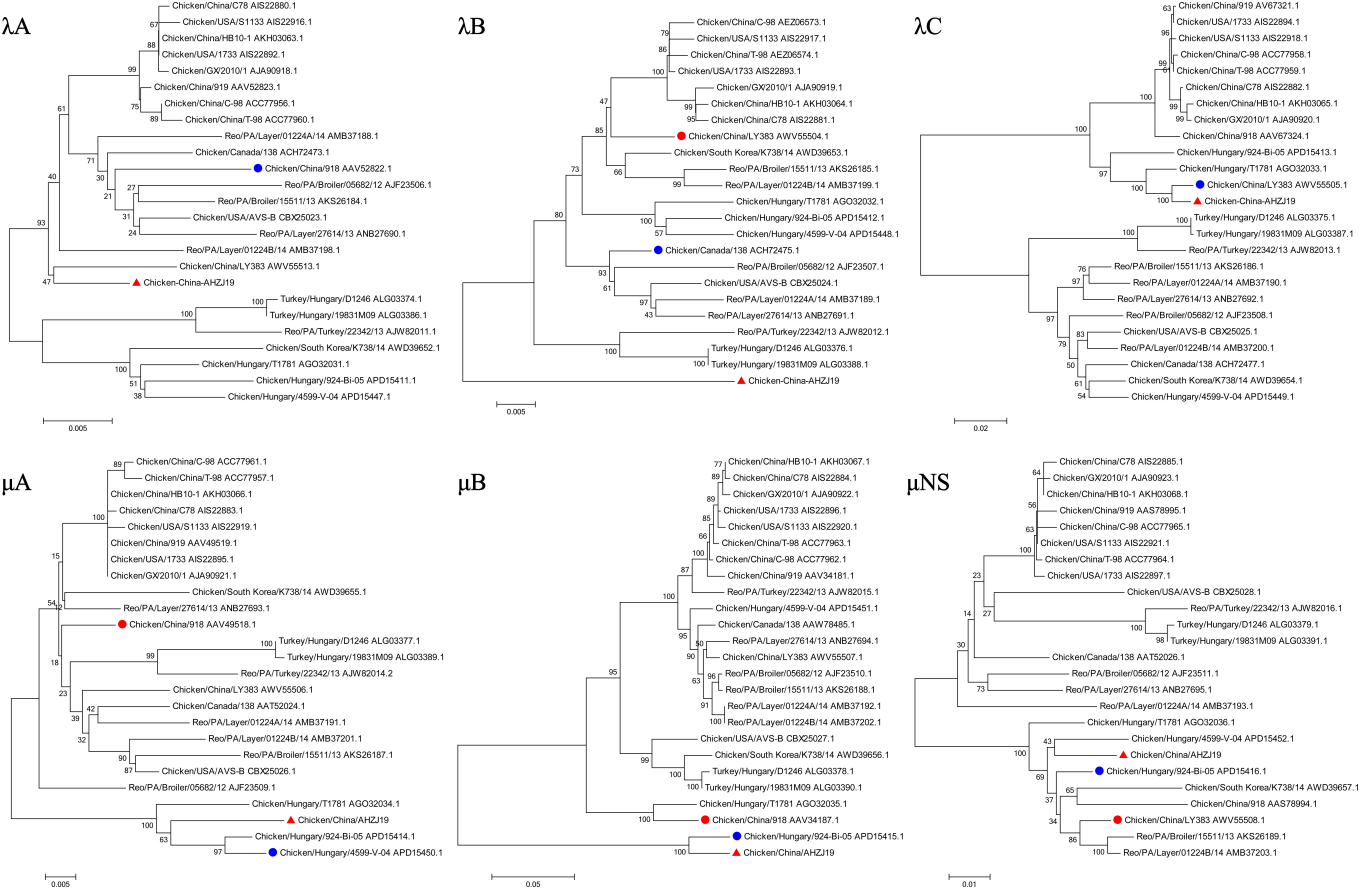
Phylogenetic trees of AHZJ19 and reference strains based on aa sequences of λA, λB, λC, μA, μB, and μNS proteins. The phylogenetic tree was constructed using the neighbor-joining method (1,000 bootstraps) with MEGA6. The AHZJ19 strain isolated in Anhui in 2019 is indicated by the red triangle. The strain sharing the highest homology with AHZJ19 in the world and in China is indicated by the blue and red circle, respectively.

**Figure 4:**
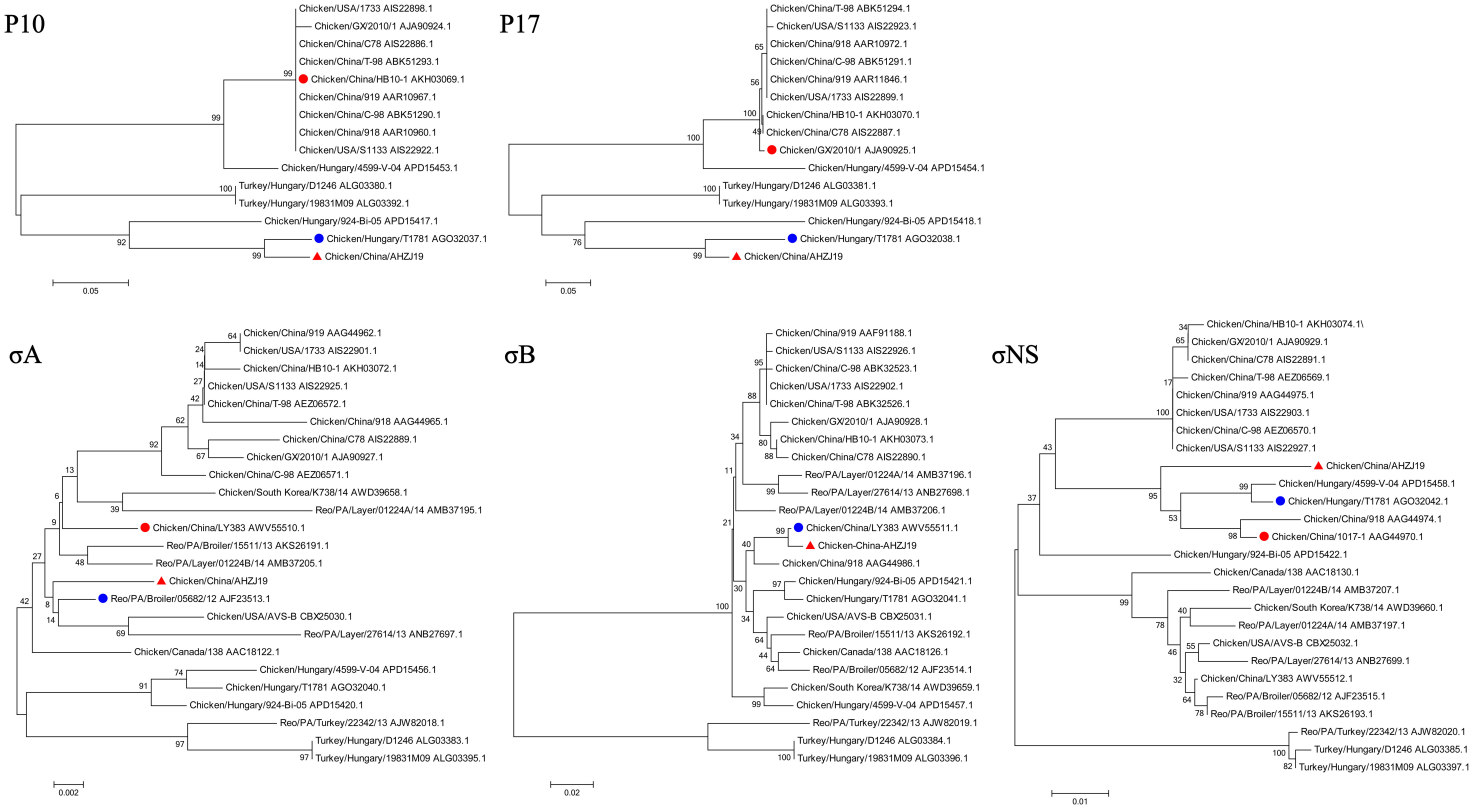
Phylogenetic trees of AHZJ19 and reference strains based on aa sequences of P10, P17, σA, σB, and σNS proteins. The phylogenetic tree was constructed using the neighbor-joining method (1,000 bootstraps) with MEGA6. The AHZJ19 strain isolated in Anhui in 2019 is indicated by the red triangle. The strain sharing the highest homology with AHZJ19 in the world and in China is indicated by the blue and red circle, respectively.

**Figure 5:**
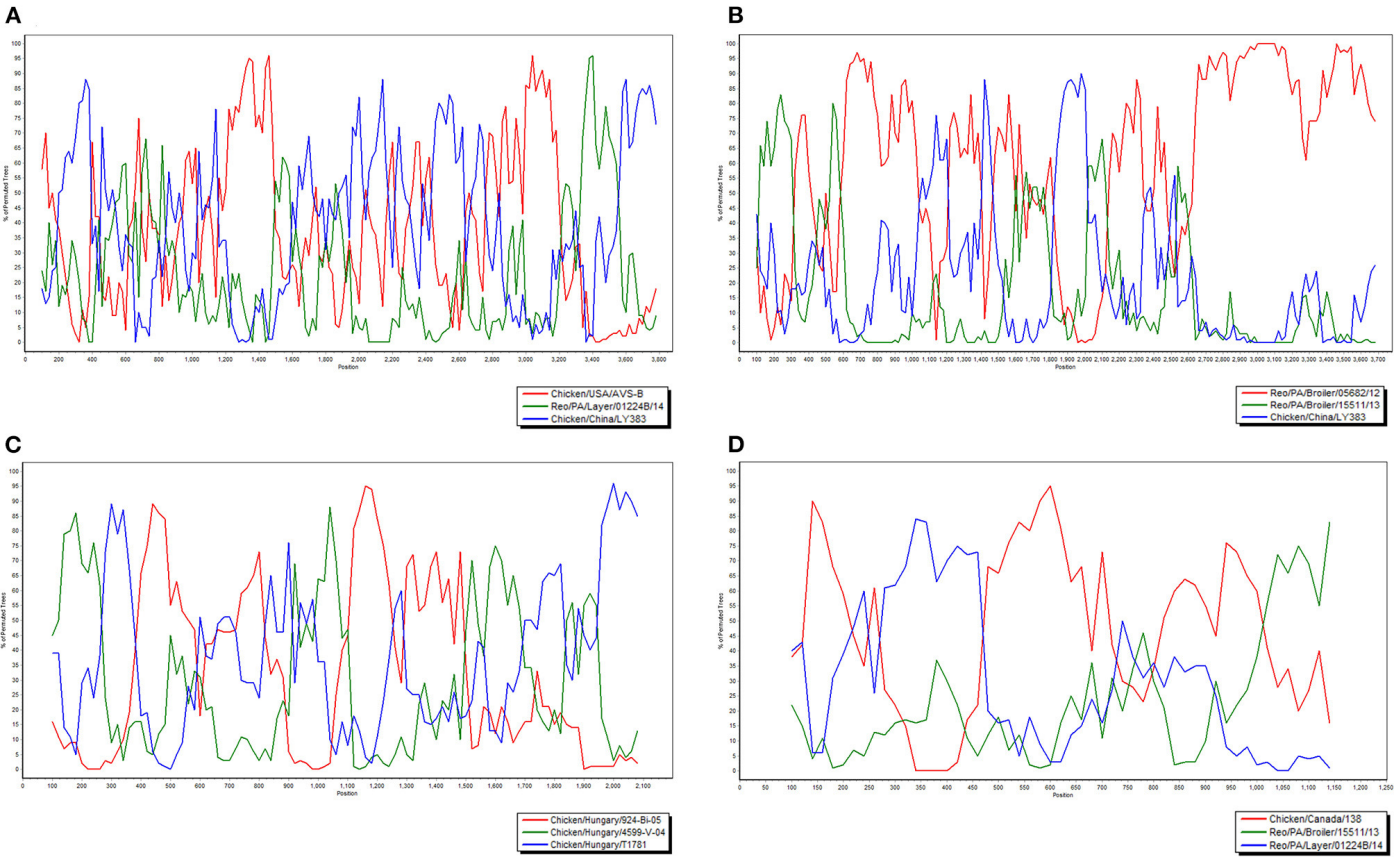
Bootscan analysis of AHZJ19 for detecting intra-segmental recombination. The Bootscan analysis was constructed using the neighbor-joining method (a Kimura two-parameter applied and 100 replicates) with Simplot program version 3.5.1. Query-Chicken/China/AHZJ19; **(A)** λA, **(B)** λB, **(C)** μA, and **(D)** σA.

To investigate the genome characteristics of AHZJ19, the high-throughput sequencing by Illumina sequencing technology was performed. The sequencing data revealed that the genome size of AHZJ19 is about 23,230 bp with the standard genome structure of ARV, which codes 12 major proteins. To elucidate the molecular characteristics of AHZJ19, the aa sequences of the 12 major proteins of the isolate were compared with the aa sequences of the reference ARV strains. As shown in Table 4, according to the aa BLAST of the 12 proteins, the σC protein of AHZJ19 had the highest homology (95.0%) with that of the American strain 99847 and only 53.2% homology with that of the vaccine strain S1133 and 1733 (data not shown). Notably, the highest homology of the σC protein between AHZJ19 and Chinese strains previously reported was only 82.8% (strain SD18). Except for σC protein, as described in Table 4, the highest homology strain with AHZJ19 for λA, λB, λC, μA, μB, μNS, P10, P17, σA, σB, and σNS was the China 919, Canada 138, China LY383, Hungary 4599-V-04, Hungary 924-Bi-05, Hungary 924-Bi-05, Hungary T1781, Hungary T1781, USA 05682/12, China LY383 and Hungary T1781, respectively. Notably, for λA, λB, λC, μA, μB, μNS, P10, P17, σA, σB, and σNS, AHZJ19 shared the highest homology with the Chinese strain 919, strain LY383, strain LY383, strain 918, strain 918, strain LY383, strain HB10-1, strain GX/2010/1, strain LY383, strain LY383, and strain 1017-1, respectively (Table 4). These data allow us to speculate about genetic reassortment of the AHZJ19 isolate and the relatively high genetic distance between the recombinant and parental strains was potentially due to the unsequenced intermediate strains. The phylogenetic tree analysis for these proteins further revealed that AHZJ19 strain might have originated from a recombination of Hungarian strains, North American strains, and Chinese strains (Figures 2–4). Moreover, according to the Bootscan analysis within Simplot program, recombination detection further highlighted the possibility of intra-segmental recombination of the λA, λB, μA, and σA genes of AHZJ19 with reference strains. The λA gene of AHZJ19 may have recombined with USA/AVS-B (GenBank accession number: FR694191.1), Reo/PA/Layer/01224B/14 (KT428308.1), and China/LY383 (MF183221.1) strains (Figure 5A) and the λB gene may have recombined with Reo/PA/Broiler/05682/12 (KM877326.1), Reo/PA/Broiler/15511/13 (KP731612.1), and China/LY383 (MF183212.1) strains (Figure 5B). Similarly, there is evidence that the μA gene may have recombined with Hungary/924-Bi-05 (KX398265.1), Hungary/4599-V-04 (KX398295.1), and Hungary/T1781 (KC865789.1) strains (Figure 5C); besides, it appears that the σA gene recombined with Canada/138 (AF059717.1), Reo/PA/Broiler/15511/13 (KP731618.1), and Reo/PA/Layer/01224B/14 (KT428315.1) strains (Figure 5D). Taken together, the generation of AHZJ19 reflected the genomic reassortment and intra-segmental recombination between previously identified ARV strains throughout the world.

**Table 4:** Genomic characteristics of AHZJ19.

**Gene segments**	**Gene length/bp**	**Encoded protein**	**Protein length/bp**	**Protein length/aa**	**aa homology with S1133 strain /%**	**The strain with the highest aa homology in the world and its homology/%**	**The strain with the highest aa homology in China and its homology/%**
L1	3,928	λA	3,882	1,294	98.5	Chicken/China/919, 98.7	Chicken/China/919, 98.7
L2	3,822	λB	3,780	1,260	93.6	Chicken/Canada/138, 94.2	Chicken/China/LY383, 94.0
L3	3,876	λC	3,858	1,286	93.0	Chicken/China/LY383, 98.4	Chicken/China/LY383, 98.4
M1	2,247	μA	2,199	733	93.3	Chicken/Hungary/4599-V-04, 97.0	Chicken/China/918, 94.0
M2	2,066	μB	2,031	677	63.7	Chicken/Hungary/924-Bi-05, 95.0	Chicken/China/918, 72.7
M3	1,990	μNS	1,908	636	92.3	Chicken/Hungary/924-Bi-05, 97.0	Chicken/China/LY383, 96.7
S1	1,620	P10	300	100	68.7	Chicken/Hungary/T1781, 93.9	Chicken/China/HB10-1, 70.7
		P17	441	147	61.9	Chicken/Hungary/T1781, 89.0	Chicken/GX/2010/1, 64.1
		σC	981	327	53.2	Chicken/USA/99847, 95.0	Chicken/China/SD18, 82.8
S2	1,312	σA	1,251	417	98.3	Reo/PA/Broiler/05682/12, 99.0	Chicken/China/LY383, 98.6
S3	1,195	σB	1,104	368	94.6	Chicken/China/LY383, 99.2	Chicken/China/LY383, 99.2
S4	1,174	σNS	1,104	368	93.2	Chicken/Hungary/T1781, 95.6	Chicken/China/1017-1, 95.6

**Figure 6:**
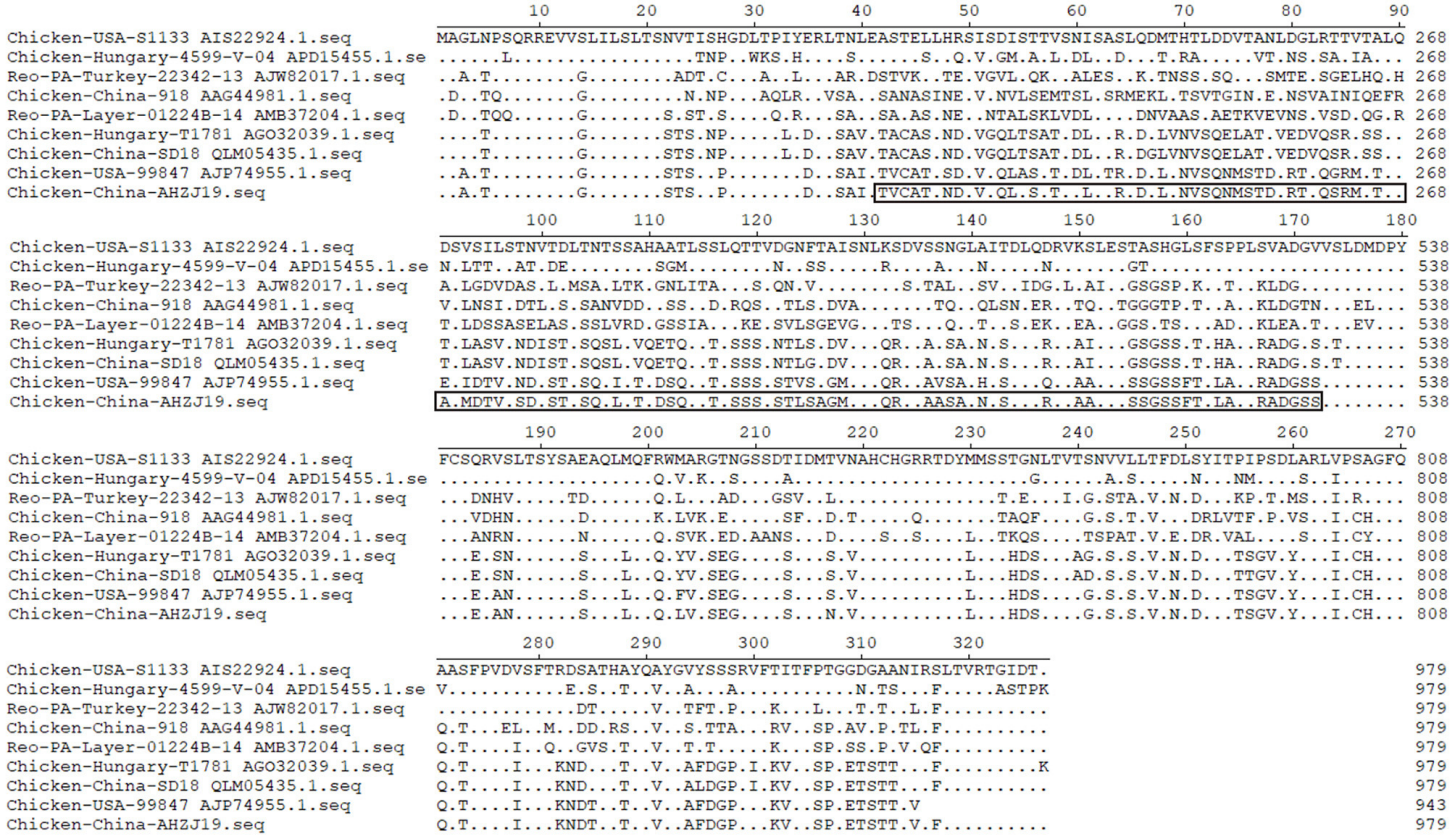
Alignment of AHZJ19 and eight reference strains based on σC protein. The alignment was constructed using the ClustalW methods in Megalign program included in the Lasergene 7.0 software program.

Of note, based on the σC protein of AHZJ19 in the phylogenetic tree and a relevant study (34), AHZJ19 was clustered into Group IV (Figure 2). Although Chinese strain SD18 and Hungary strain T1781 also belonged to Group IV, the homology of the σC protein between AHZJ19 and Chinese strain SD18 or Hungary strain T1781 was only about 80%. Regardless of the genetic variability of the σC protein, from an evolution perspective, the strains had diverged and evolved independently quite a long time ago and in-depth analysis depends on more unsampled or unsequenced strains. Notably, the homology of the σC protein between Chinese strain SD18 and Hungary strain T1781 reached 98.5%, indicating that Group IV could be further divided into two subgroups, AHZJ19-like and SD18-like. As a dominant antigenic protein and the viral cell attachment protein, σC protein can efficiently induce the specific neutralizing antibodies against ARV (2, 6). However, σC protein is the highest variant protein among the 12 proteins and the hypervariable regions of σC protein are mainly located in 1–122 and 196–326 aa residues (35). In comparison with ARV reference strains, multiple mutations were identified in the σC protein between the different groups and AHZJ19 as shown in Figure 6. Moreover, six antigenic sites (74–76 aa, 78–82 aa, 86–88 aa, 93–95 aa, 109–112 aa, and 125–128 aa) of the σC protein were variant between AHZJ9 and strain SD18. As vaccine strains S1133 and 1733 have been widely used in the poultry industry, the low homology of the σC protein of AHZJ19 with that of these vaccine strains highlights that current vaccines might not provide efficient protection against the novel ARV isolate AHZJ19.

In summary, this is the first demonstration of a novel ARV strain AHZJ19 with the unique gene constellation possibly originated from the recombination of Hungarian strains, North American strains, and Chinese strains. Although the σC protein sequences of almost all ARV strains can be obtained from GenBank, the sequences of other proteins of most strains have not been deposited in GenBank yet. In addition, coupled with the lack of some unsampled or unsequenced strains, the above factors are the limitations of this study for recombination analysis. Thus, our study shows that the σC protein of AHZJ19 is closely related to that of the American strain 99847, but significantly different from that of the vaccine strains S1133, 1733, and Chinese strains previously reported, highlighting the inefficient protection of these vaccines against such ARV variant. Notably, lots of ARV mutants have recently emerged in broiler flocks globally, and some of them have become prevalent in different regions, such as North America, which presents a challenge to the current vaccine strategies for ARV. An in-depth recombination analysis of the novel AHZJ19 ARV isolate is necessary and will certainly be performed once the complete genome sequences of all strains involved in this study (the American strain 99847 and Chinese strain SD18) become publicly available. Therefore, it is critical to study on the molecular epidemiology for these ARV variants for better controlling of the diseases caused by ARV. Of course, the pathogenesis and the mechanism of the recombination of the novel ARV isolate AHZJ19 need to be further elucidated.

## Data Availability Statement

The original contributions presented in the study are included in the article/supplementary material, further inquiries can be directed to the corresponding author/s.

## Ethics Statement

The animal study was reviewed and approved by the Animal Care Committee at Yangzhou University in China.

## Author Contributions

HS conceived and designed the experiments. JZ, TL, WW, and QX performed the experiments. JZ, TL, and HS analyzed the data. ZW and AQ contributed reagents, materials, and analysis tools. JZ, TL, HS, and JY contributed to the writing of the manuscript. All authors have read and approved the final manuscript.

## Funding

This study was supported by the National Key Research and Development (R&D) Plan (2018YFD0500106), the Key Laboratory of Prevention and Control of Biological Hazard Factors (Animal Origin) for Agrifood Safety and Quality (26116120), the Research Foundation for Talented Scholars in Yangzhou University, and the Priority Academic Program Development of Jiangsu Higher Education Institutions.

## Conflict of Interest

The authors declare that the research was conducted in the absence of any commercial or financial relationships that could be construed as a potential conflict of interest.

## Publisher's Note

All claims expressed in this article are solely those of the authors and do not necessarily represent those of their affiliated organizations, or those of the publisher, the editors and the reviewers. Any product that may be evaluated in this article, or claim that may be made by its manufacturer, is not guaranteed or endorsed by the publisher.
